# A Novel Gum Paint Formulation Derived From Licorice and Triphala: Characteristics and Clinical Significance for Improved Oral Health

**DOI:** 10.7759/cureus.63940

**Published:** 2024-07-06

**Authors:** Neha Kannan, Gheena S, Pratibha Ramani, Rajeshkumar Shanmugam, Karthikeyan Ramalingam

**Affiliations:** 1 Oral and Maxillofacial Pathology, Saveetha Dental College and Hospitals, Saveetha Institute of Medical and Technical Sciences, Saveetha University, Chennai, IND; 2 Nanobiomedicine Lab, Centre for Global Health Research, Saveetha Medical College and Hospital, Saveetha Institute of Medical and Technical Sciences, Saveetha University, Chennai, IND

**Keywords:** green synthesis, anti-fungal, antimicrobial activity, licorice, triphala, gum paint

## Abstract

Background: The clinical use of antimicrobial agents for managing aphthous ulcers and periodontal diseases has long been a subject of intensive research by numerous investigators. As concerns over the side effects and antibiotic resistance associated with conventional therapies persist, there has been a concerted effort to explore alternative medicinal approaches. In line with this objective, our study introduces a novel herbal gum paint designed specifically to address the therapeutic needs of individuals suffering from oral ulcers and periodontal diseases.

Materials and methods: The herbal formulation utilized in our study was prepared using extracts derived from Licorice (*Glycyrrhiza glabra*) and Triphala, a combination of three fruits: *Emblica officinalis*, *Terminalia chebula*, and *Terminalia belerica*. These ingredients were selected based on their documented medicinal properties. The preparation process involved extraction and formulation techniques optimized for maximum efficacy. Antimicrobial activity was assessed using the bacterial culture method, where the formulation’s ability to inhibit the growth of specific bacterial strains relevant to oral health was tested. Meanwhile, cytotoxicity was evaluated using the Brine Shrimp Assay method. Statistical analysis was conducted using a one-way analysis of variance (ANOVA) and Tukey post hoc test to validate the significance of our findings with statistical significance set at p<0.05.

Results: The formulation exhibited significant activity against microbes when compared to the control. The cytotoxic activity was present at a concentration of 60 and 80µL, which indicated safe usage within specified concentration ranges, highlighting its potential for clinical application without adverse effects on biological systems. Statistically significant differences were obtained between the antimicrobial activity of the formulated gum paint and the commercial gum paint against *Candida* *albicans* species at 25 µL and 80 µL (p=0.00).

Conclusion: The study underscores the promising therapeutic potential of the herbal gum paint developed in this research. By harnessing the natural antimicrobial and anti-inflammatory properties of Licorice and Triphala, the formulated gum paint showed efficacy against *C. albicans. *These findings contribute to the growing body of evidence supporting the integration of herbal remedies into mainstream oral healthcare practices. Future investigations could further elucidate the mechanisms underlying its therapeutic actions and explore its broader clinical applications in diverse patient populations.

## Introduction

Oral aphthous ulcers, commonly known as canker sores, are painful, inflamed lesions that develop on the oral mucosa [1]. These ulcers can cause significant discomfort and interfere with eating, speaking, and oral hygiene. Oral aphthous ulcerations can manifest independently or as a complication associated with a variety of different disease processes, ranging from nutritional deficiencies and gastrointestinal disorders to systemic autoimmune conditions. Recurrent aphthous stomatitis is a condition in which recurrence is frequent [2]. Several microorganisms have been implicated in the progression of aphthous lesions, suggesting a complex interplay between the host's immune system and microbial agents. The involvement of bacteria, viruses, and possibly fungi points to a multifactorial aetiology [2].

Effective treatment for aphthous ulcers should focus on pain relief, reduction of the microbial load and promoting healing without recurrence. Topical treatments, such as corticosteroid ointments like hydrocortisone or triamcinolone acetonide, can reduce inflammation and pain, while antiseptic gels containing chlorhexidine gluconate help decrease bacterial load and prevent secondary infections. Lidocaine or benzocaine gels provide temporary pain relief by numbing the affected area. However, some treatments may have side effects, such as oral corticosteroids potentially can cause systemic effects like increased susceptibility to infections or gastrointestinal issues, and topical anaesthetics sometimes can lead to allergic reactions [2,3].

Periodontal diseases are complex, multifactorial, and polymicrobial infections that result in the destruction of the tissues supporting the teeth, including the gingiva, periodontal ligament, and alveolar bone. These diseases are primarily caused by pathogenic bacterial microflora that colonize the oral cavity, forming a biofilm on the surfaces of the teeth. The pathogenic bacteria in this biofilm trigger an inflammatory response in the host, and in susceptible individuals, this response can lead to the progressive destruction of the periodontal tissues. The interaction between the bacterial pathogens and the host's immune response is critical in the progression of periodontal diseases [4].

Effective prevention and management of periodontal diseases primarily involve the eradication of the microbial biofilm. This can be achieved through professional dental care and consistent self-care practices. Professional cleaning, which typically occurs on a quarterly or bi-annual basis, is essential for the removal of plaque and calculus that cannot be eliminated by routine brushing and flossing [5]. In addition to professional care, regular self-performed oral hygiene is crucial in maintaining periodontal health. This includes daily brushing, flossing, and the use of antimicrobial mouth rinses to control plaque formation and bacterial proliferation. However, the motivation and dexterity required to maintain optimal oral hygiene can be challenging for many individuals. Factors such as age, physical limitations, cognitive impairments, and a lack of understanding of proper oral care techniques can all contribute to suboptimal oral hygiene practices [6].

Further, antibiotic therapy, involving local or systemic antibiotics such as doxycycline, amoxicillin, or metronidazole, is commonly used to reduce bacterial infection. Overall, the prevention and management of aphthous ulcers and periodontal diseases require a comprehensive approach that includes both professional intervention and effective self-care practices [7].

To improve the efficacy of routine oral hygiene measures, many chemotherapeutic agents have been developed to control bacterial plaque. In the last few decades, there has been a growing interest in the development of plant-based medicines with antibacterial and anti-inflammatory properties for the treatment of oral diseases. This will help to mitigate the risks associated with the widespread misuse of chemotherapeutic agents, which cause microbial drug resistance [8].

There is a substantial amount of research supporting the use of herbs in the prevention and treatment of human ailments [2,3,9]. Licorice and Triphala are among the phytochemicals that have been an integral part of Ayurvedic treatments for diseases of the oral cavity. In Chinese medicine, Licorice is a common herb. It is a sweet, moist, soothing herb of the *Glycyrrhiza *genus native to the Mediterranean and Asia. Licorice contains many secondary metabolites that have been implicated in several health advantages. Several disorders, including cancer, TB, atherosclerosis, gastric ulcers, immunodeficiency, hepatitis, and bacterial infections, have been reported to respond favourably to secondary metabolites found in Licorice roots. The advantages of Licorice in treating oral illnesses have recently attracted a lot of attention. The effectiveness of Licorice and its metabolites in preventing and treating a wide range of oral disorders, including periodontal disease, candidiasis, aphthous ulcers, and even life-threatening conditions like oral cancer, has been investigated in a variety of trials. Glycyrrhizin (GL), one of the flavonoids, is the primary ingredient that actively works to lessen illness symptoms [6,10].

Indian gooseberry (Amalaki, *Emblica officinalis Gaertn.*), Bibhitaki (*Terminalia bellirica*), and Haritaki (*Terminalia chebula Retz.*) are three herbal plants whose fruits combine to form Triphala, known for their ability to inhibit various microbes and enhance oral flora. Triphala possesses multiple biological activities, including anti-inflammatory, antibacterial, antifungal, antiviral, and anti-malarial properties. It has been shown to reduce collagenase activity, demonstrating an inhibitory effect against matrix metalloproteinase 9 (MMP9) [11].

However, as far as we know, there is limited information comparing and evaluating the combined benefits of Triphala and Licorice for the management of oral ulcers and periodontitis. This study aimed to assess the antimicrobial properties and cytotoxicity of Licorice and Triphala extracts in an in vitro setting. The primary objective of the study involved the formulation of a gum paint with an obtained extract. The secondary objective was to assess the antimicrobial and cytotoxicity of the formulated gum pain which can potentially aid in the treatment of periodontitis, oral ulcers and halitosis.

## Materials and methods

Preparation of extract

To prepare the extract, a precise method was followed to ensure consistency and efficacy. Initially, 1 gram of Licorice powder and 1 gram of Triphala powder were thoroughly mixed. This mixture was then combined with 100 millilitres of distilled water in a suitable container. The solution was brought to a boil, ensuring that the active components of the powders were effectively extracted into the water.

Following the boiling process, the solution underwent double filtration to remove any solid residues. This step was crucial to obtain a clear liquid free of particulate matter. The filtrate was then boiled again until the volume was reduced to approximately 15 millilitres. This reduction process concentrated the extract, enhancing its potency.

To the concentrated extract, glycerol was added. Glycerol serves multiple purposes: it acts as a humectant, maintaining moisture in the extract, and it also provides a slightly sweet taste that can help improve the palatability of the final product. After adding glycerol, the mixture was subjected to centrifugation. Centrifugation helps to further purify the extract by separating any remaining fine particulates and ensuring a homogeneous solution.

**Figure 1:**
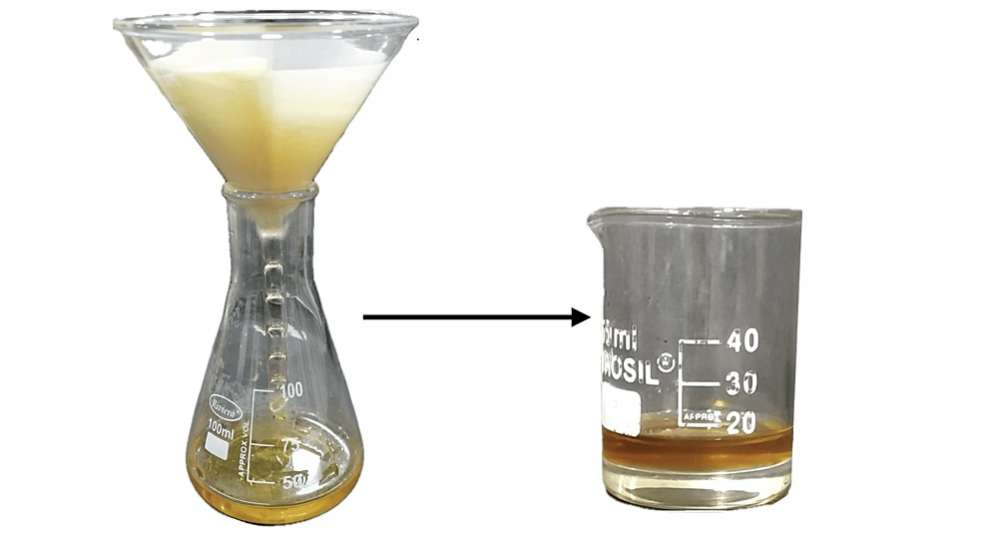
Extract preparation Double filtration of the extract to remove any solid residues subsequent to which formulated gum paint was obtained from Licorice and Triphala.

For flavour enhancement, peppermint and lemon essential oils were added to the extract. These essential oils not only improve the taste and smell of the preparation but also contribute additional antimicrobial and anti-inflammatory properties, making the extract more beneficial for oral health applications. By following this detailed preparation method, a potent and palatable extract of Licorice and Triphala was obtained (Figure 1).

Antimicrobial activity using bacterial culture method

To evaluate the antimicrobial activity of the extract, a bacterial culture method was employed. Fresh bacterial cultures were prepared using a Hi-Veg broth medium (HiMedia Laboratories Pvt. Ltd., Mumbai, India) to ensure optimal growth conditions. Specific cultures of *Candida albicans*, *Streptococcus mutans*, *Staphylococcus aureus*, and *Enterococcus faecalis* were used for this assessment. Each bacterial culture was inoculated with 10 microliters of the respective organism and incubated for 18 hours to allow for adequate growth and establishment.

Following the incubation period, a nutrient agar medium was prepared as the growth substrate for the bacteria. Small wells, each with a diameter of 5 millimetres, were created in the agar to accommodate different concentrations of the extract, ranging from 25 to 75 micrograms per millilitre. These wells were carefully filled with the extract solutions. Additionally, antibiotic disks soaked in a commercial antimicrobial agent, specifically commercial gum paint, were used as positive controls to provide a benchmark for the extract's efficacy.

The prepared agar plates, containing both the extract-filled wells and the control antibiotic disks, were then incubated at 37 degrees Celsius for another 18 hours. This incubation period allowed the bacteria to grow and interact with the antimicrobial agents present in the wells. After the incubation, the zones of inhibition areas where bacterial growth was prevented were measured around each well. These measurements provided a quantitative assessment of the extract's antimicrobial effectiveness against the selected bacterial strains. This methodical approach ensured a comprehensive evaluation of the extract's potential in combating microbial infections associated with oral health issues.

Cytotoxic activity using the brine shrimp assay method

Salt Water Preparation

To prepare the salt water necessary for the assay, 200 millilitres of distilled water were used to dissolve 2 grams of iodine-free salt. This solution provided the required saline environment for the brine shrimp.

Procedure

Saline water was dispensed into six-well ELISA (enzyme-linked immunosorbent assay) plates, with each well receiving 10 to 12 millilitres of the prepared saline solution. Ten brine shrimp nauplii were then carefully added to each well. Different concentrations of the extract, specifically 5, 10, 20, 40, 60, and 80 microliters, were introduced into the respective wells to test a range of dosages.

The ELISA plates containing the brine shrimp and the various concentrations of the extract were incubated for 24 hours. This incubation period allowed for the interaction between the nauplii and the extract, enabling the assessment of any cytotoxic effects.

Evaluation

After the 24-hour incubation period, the plates were carefully examined to count the number of surviving nauplii in each well. The number of living brine shrimp was recorded and used to calculate the cytotoxicity. The formula used for this calculation was $\frac{100}{\text{{number of dead nauplii}} + \text{{number of live nauplii}}}$.

This methodical approach provided a quantitative measure of the extract's cytotoxic activity, allowing for an assessment of its potential toxicity. By using a range of concentrations, the assay could determine the dose-dependent effects of the extract, contributing valuable information to its safety profile.

Statistical analysis

The tests were conducted in triplicate, and the resulting values were recorded in a Microsoft Excel sheet (Microsoft® Corp., Redmond, WA, USA). Statistical analysis was performed using Statistical Package for the Social Sciences (IBM SPSS Statistics for Windows, IBM Corp., Version 26.0, Armonk, NY). A one-way analysis of variance (ANOVA) test was employed to compare the means of antibiotic inhibition between the control and various concentrations of the gum paint. Tukey's post hoc test was then conducted for multiple comparisons. This experimental in vitro study has been approved by the scientific review board with the SRB number SRB/SDC/FACULTY/24/OPATH/008.

## Results

Antimicrobial activity

The gum paint concentrations of 25 µL, 50 µL, and 80 µL effectively reduced the bacterial counts for *S. aureus, S. mutans, *and* E.faecalis*. The established commercial control gum paint was found to be better against *S. aureus, S. mutans, *and* E. faecalis* except in the case of *C. albicans*. For *C. albicans*, the results were mixed, with 25 µL and 80 µL concentrations showing significant differences, while 50 µL did not. Therefore, for *C. albicans*, the formulated gum paint showed effectiveness (Table 1).

**Table 1:** Antimicrobial activity The table shows the statistical differences in the antimicrobial activity between the formulated gum paint and commercial control against *Staphylococcus aureus*, *Streptococcus mutans*, *Enterococcus faecalis* and *Candida albicans* in different concentrations of 25µL, 50µL and 80µL respectively. Tests employed one-way analysis of variance (ANOVA) and Tukey’s post hoc test. Statistical significance set at p <0.05.

Bacterial Species	Comparison	Mean Difference	Standard Error	p-value	Interpretation
Staphylococcus aureus	Antibiotic control vs. 25 µL	10	0.577	0.00*	Significant reduction in *S. aureus*
	Antibiotic control vs. 50 µL	8.333	0.577	0.00*	Significant reduction in *S. aureus*
	Antibiotic control vs. 80 µL	5.667	0.577	0.00*	Significant reduction in *S. aureus*
Streptococcus mutans	Antibiotic control vs. 25 µL	7.667	0.577	0.00*	Significant reduction in *S. mutans*
	Antibiotic control vs. 50 µL	4.333	0.577	0.00*	Significant reduction in *S. mutans*
	Antibiotic control vs. 80 µL	2	0.577	0.00	Significant reduction in *S. mutans*
Enterococcus faecalis	Antibiotic control vs. 25 µL	8.333	0.408	0.00*	Significant reduction in *E. faecalis*
	Antibiotic control vs. 50 µL	5.667	0.408	0.00*	Significant reduction in *E. faecalis*
	Antibiotic control vs. 80 µL	2.333	0.408	0.00*	Significant reduction in *E. faecalis*
Candida albicans	Antibiotic control vs. 25 µL	1.667	0.408	0.00*	Significant reduction in *C. albicans*
	Antibiotic control vs. 50 µL	0	0.408	1	No significant difference in *C. albicans*
	Antibiotic control vs. 80 µL	-1.667	0.408	0.00*	Significant increase in *C. albicans*

**Figure 2:**
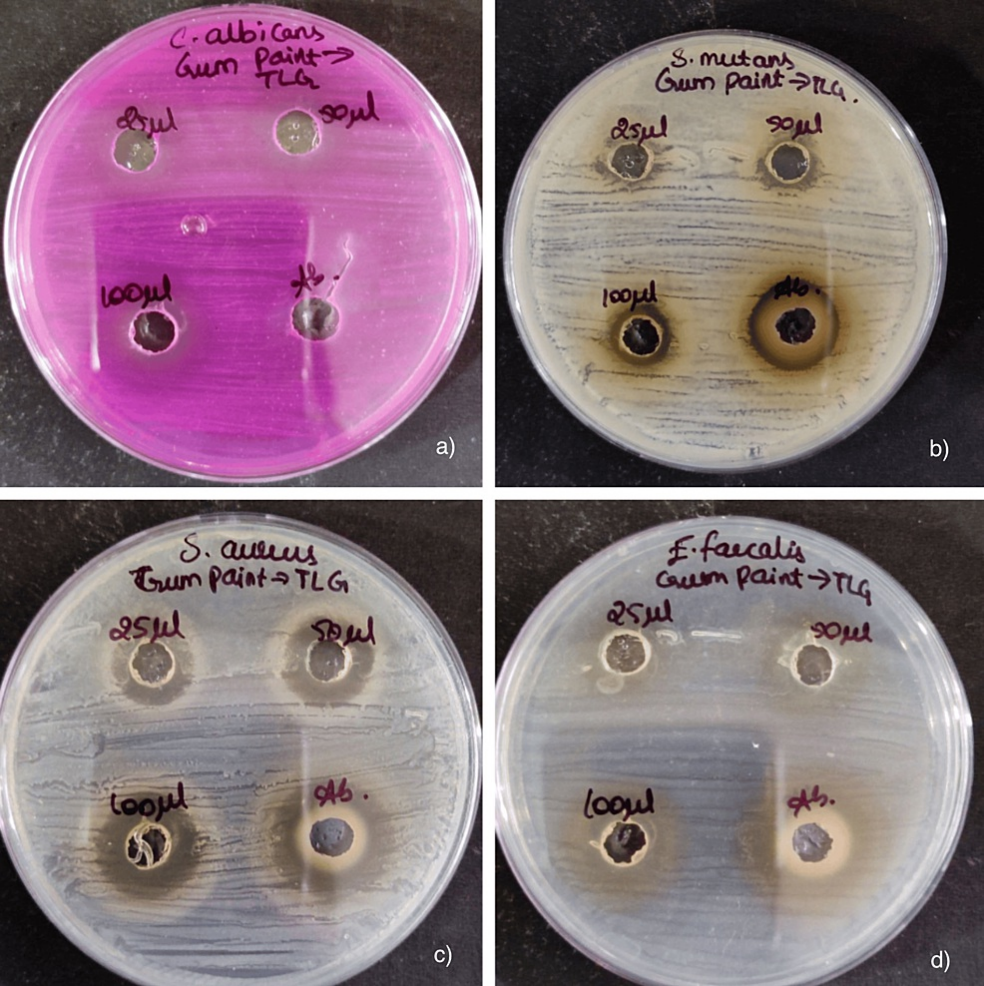
Zone of inhibition of the formulated gum paint Antimicrobial activity (zone of inhibition) of the formulated gum paint in different concentrations against (a) *Candida albicans*, (b) *Streptococcus mutans*, (c) *Staphylococcus aureus *and (d) *Enterococcus faecalis* respectively in nutrient agar medium.

**Figure 3:**
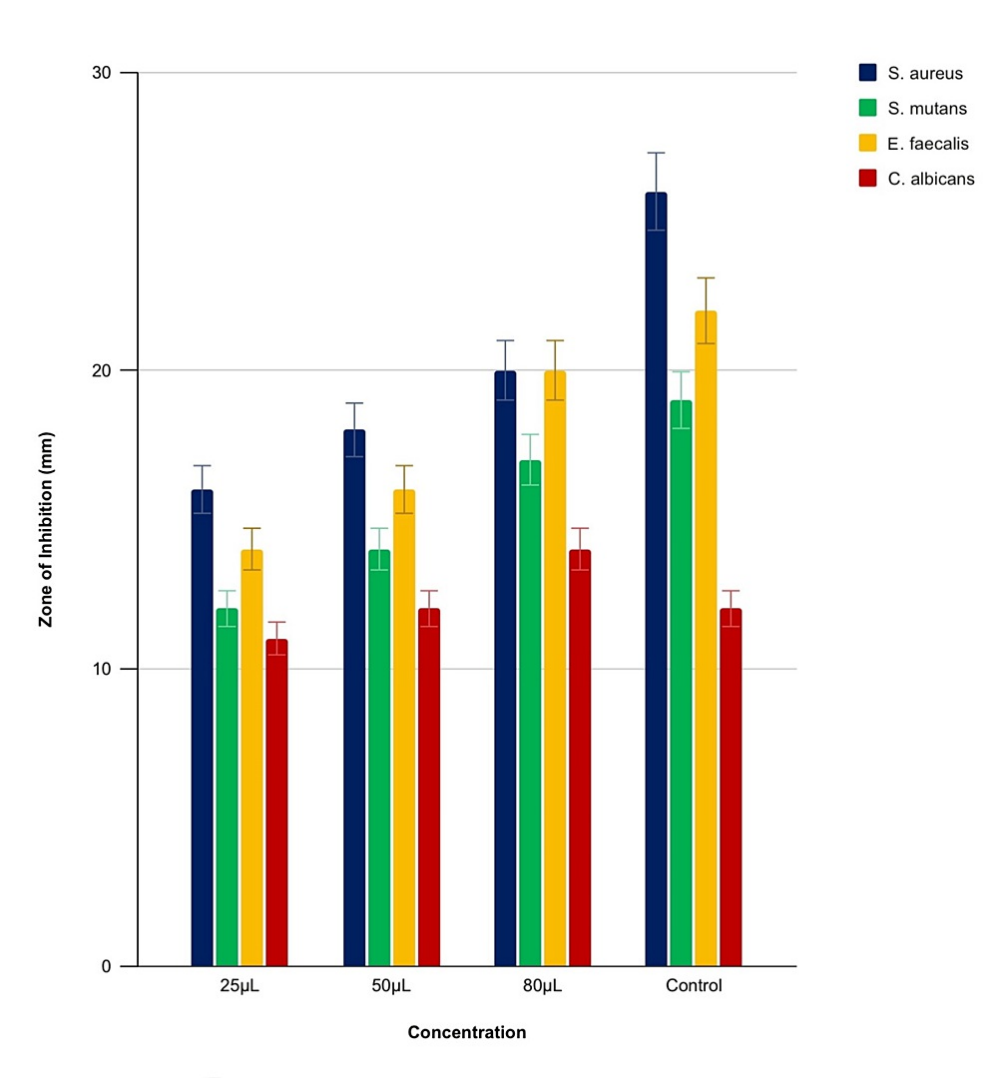
Antimicrobial activity of the gum paint and the established commercial gum paint (control) The antimicrobial activity of the gum paint and the established commercial gum paint (control) showed the highest activity against C*andida albicans*. The antimicrobial activity against *Staphylococcus aureus*, *Streptococcus mutans* and *Enterococcus faecalis *are also depicted.

These results indicate that the gum paint has a notable antimicrobial effect, particularly at higher concentrations, though it generally falls short of the efficacy exhibited by the antibiotic control (Figures 2, 3). Specifically, the gum paint showed higher activity against *C. albicans* at 80 μL compared to the established commercial gum paint (control). At other concentrations, the formulated gum paint had reduced efficacy. The highest activity was observed against *S. aureus* almost equal to the control, followed by *E. faecalis *(Figure 3).

Cytotoxicity

**Figure 4:**
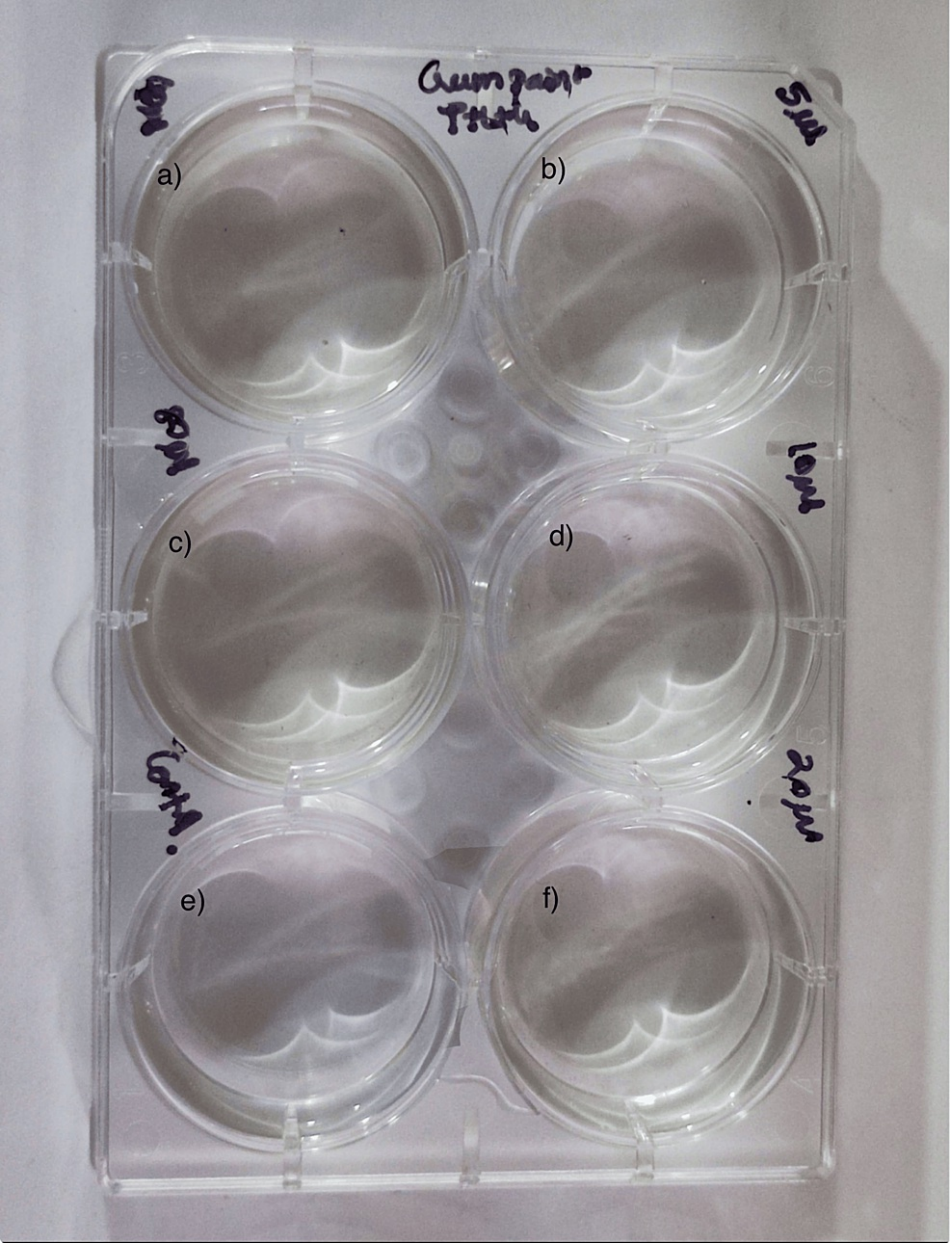
Evaluation of cytotoxic activity Cytotoxic activity exhibited by gum paint in different concentrations at (a) 40 µL, (b) 5 µL, (c) 60 µL, (d) 10 µL, (e) 80 µL and (f) 20 µL respectively.

**Figure 5:**
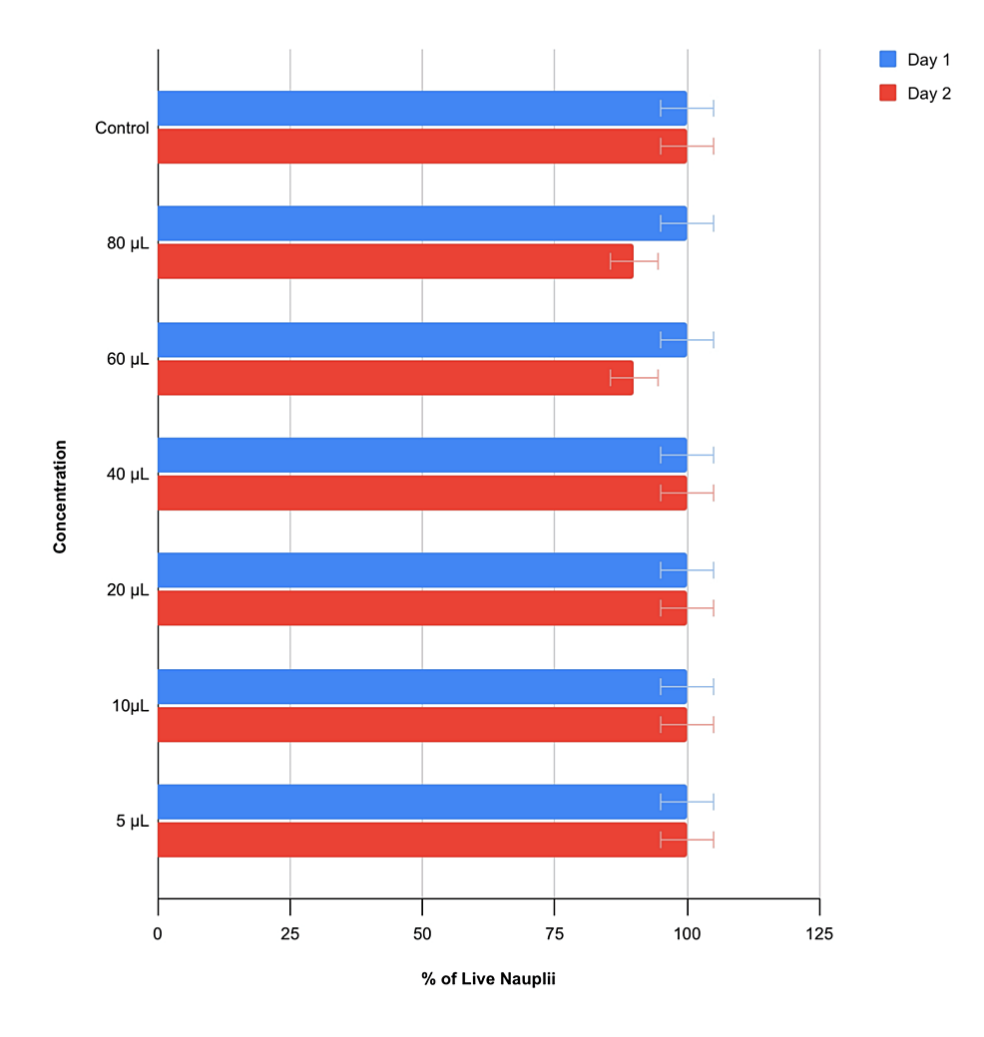
Cytotoxicity in terms of the percentage of live nauplii at different concentrations Graph representing the % of live nauplii in different concentrations exhibited on days 1 and 2. On day 2, the highest cytotoxicity was observed at a concentration of 60 and 80 μL.

The cytotoxic test using brine shrimp nauplii was conducted over a time period of two days (48 hours) to evaluate the effects of different concentrations of the extract. On day 1, all concentrations, including 5 µL, 10 µL, 20 µL, 40 µL, 60 µL, and 80 µL, as well as the control, showed a 100% survival rate of the nauplii. By day 2, the survival rate remained at 100% for the 5 µL, 10 µL, 20 µL and 40 µL concentrations, as well as for the control group. However, at 60 µL and 80 µL concentrations, the survival rate decreased to 90%. These results suggest a concentration-dependent increase in cytotoxicity of the extract, particularly evident on the second day of the assay (Table 2, Figures 4, 5).

**Table 2:** Cytotoxicity of the gum paint The results of the cytotoxic activity of the formulated gum paint assessed against the established commercial gum paint were calculated twice at 24 hours and 48 hours respectively.

Concentrations	24 Hours (% of Live Nauplii)	48 Hours (% of Live Nauplii)
5 µL	100	100
10 µL	100	100
20 µL	100	100
40 µL	100	100
60 µL	100	90
80 µL	100	90
Control	100	100

## Discussion

This in vitro experimental study represents a pioneering effort to incorporate both Triphala and Licorice into a gum paint formulation, marking a significant advancement in oral healthcare research. Previous investigations have extensively documented the therapeutic benefits of the individual components, particularly focusing on Licorice [12]. Numerous studies conducted in recent years have highlighted that the active compounds isolated from Licorice exhibit a wide array of biological activities. These compounds are known for their potent antimicrobial properties, making them effective against a variety of bacterial strains.

Our study revealed that the gum paint formulated using Licorice and Triphala extracts showed potent antimicrobial, antifungal and cytotoxic effects. These were similar to the study by many previous research. In a comprehensive study by Öztürk et al. [13], it was found that Licorice is a rich source of bioactive compounds, containing more than 20 triterpenoids and approximately 300 flavonoids. These compounds contribute significantly to Licorice's medicinal properties. The study identified several primary active substances with potent antiviral and antibacterial properties. Among these, GL stands out for its ability to inhibit viral replication and modulate immune responses. 18-glycyrrhetinic acid (GA), a derivative of GL, is known for its anti-inflammatory and antimicrobial effects. Liquiritigenin (LTG) has been shown to possess antioxidant and anti-inflammatory properties, enhancing its therapeutic potential. Licochalcone A (LCA) and licochalcone E (LCE) are notable for their strong antibacterial activities against various pathogens, making them valuable for infection control. Glabridin (GLD) is another key compound that exhibits significant antioxidant and antimicrobial activities, contributing to the overall efficacy of Licorice in medical applications. These findings underscore the multifaceted health benefits of Licorice and its potential as a natural therapeutic agent in the prevention and treatment of infectious and inflammatory diseases.

In a detailed investigation conducted by Zhou et al. [14], the antimicrobial properties of various Licorice extracts were thoroughly examined. The study focused on the effects of Licorice aqueous extract, ethanol extract, and supercritical fluid extract on a range of pathogenic microorganisms, encompassing both Gram-positive and Gram-negative bacteria. The findings revealed that these Licorice extracts significantly inhibited the growth of several harmful bacteria and fungi, including *S.* *aureus*, a common Gram-positive bacterium known for causing skin and respiratory infections; *Escherichia coli*, a Gram-negative bacterium often associated with foodborne illness; *Pseudomonas aeruginosa*, a Gram-negative bacterium known for its resistance to antibiotics and its role in hospital-acquired infections; *C. albicans*, a fungus responsible for oral and genital infections; and *Bacillus subtilis*, a Gram-positive bacterium. The study highlighted the potent antimicrobial activities of Licorice, with each type of extract-aqueous, ethanol, and supercritical fluid-demonstrating significant efficacy against these pathogens. The aqueous extract was particularly effective in inhibiting *S. aureus *and* E. coli*, while the ethanol extract showed robust activity against *P. aeruginosa *and *C. albicans*. The supercritical fluid extract exhibited the strongest overall antimicrobial effects, significantly inhibiting all tested microorganisms, including *B. subtilis*.

These results underscore the potential of Licorice extracts as powerful natural antimicrobial agents. The study suggests that Licorice could be utilized in various applications, ranging from pharmaceutical formulations to natural preservatives in food and cosmetics. The broad-spectrum activity of Licorice extracts against both bacteria and fungi positions them as valuable candidates for developing new treatments for infections, particularly in an era of increasing antibiotic resistance. This research provides a solid foundation for further exploration into the therapeutic uses of Licorice and its active components in combating infectious diseases [14].

Qiu et al.'s study [15] investigated the potential of Licorice as an intervention to mitigate MRSA (methicillin-resistant *S. aureus*) infections by targeting various virulence mechanisms. Their findings underscore Licorice's ability to inhibit the expression of key virulence genes such as SaeR and Hla, pivotal for MRSA pathogenesis. Moreover, Licorice disrupts biofilm formation and impedes the transition of *Candida *species from yeast to hyphal forms, offering promising avenues for attenuating MRSA virulence. Additionally, Licorice reduces the production of alpha-toxin and alpha-hemolysin, both potent cytotoxins, further diminishing MRSA's pathogenicity. By addressing these multiple facets of MRSA virulence, Licorice emerges as a novel therapeutic approach to enhance the management and treatment outcomes of MRSA infections, with potential benefits for patients and public health efforts alike [15].

The cytotoxic activity of flavonoids produced from the Licorice root against human oral malignant and non-malignant cells was analysed quantitatively by Ohno et al., in terms of structure-activity relationships. Licurazid demonstrated similar tumour specificity to that of liquidity and isoliquiritin, and it was about twice as hazardous to tumour cells. Their cytotoxicity is influenced by the presence of phenolic OH groups [15-17].

Omran et al.'s study [18] addressed the high morbidity and mortality associated with nosocomial infections caused by multidrug-resistant (MDR) bacterial strains. Folk medicine and ethnopharmacological data offered a range of plants with potential antimicrobial properties, including Triphala, an Ayurvedic formula composed of *Terminalia chebula Retz., Terminalia bellirica (Gaertn.) Roxb., *and* Phyllanthus emblica L*. To optimize the extraction of Triphala's bioactive constituents, various techniques were evaluated, with microwave-assisted extraction (MAE) proving the most efficient. The Triphala hydroalcoholic extract (TAE) was chemically characterized and tested alone or with carvacrol in different formulations, such as creams and nanoemulsion hydrogels, against various microorganisms. The nanoemulsion hydrogel, containing lipophilic carvacrol and hydrophilic TAE, demonstrated stability and effective antimicrobial activity, suggesting Triphala's potential as an adjuvant antimicrobial agent for treating infections.

Sahragard et al. [19] investigated the potential of natural products in modern cancer treatments, addressing resistance issues with traditional chemotherapies. Persian medicine (PM) has long used natural products for malignant and chronic diseases. This study evaluated the cytotoxic activity of Triphala, a traditional PM formulation made from *Terminalia chebula Retz., Terminalia bellirica Retz., Phyllanthus emblica L.*, and honey, on the HepG2 human liver cancer cell line. Hydroalcoholic extracts of the formulation and its components were tested using the MTT assay at various concentrations. The results showed that all concentrations of the Triphala preparation and Cisplatin significantly reduced the survival of HepG2 cells compared to the control, indicating that Triphala and its components have substantial cytotoxic activity and could be effective anticancer agents.

Prabhakar et al. [20] evaluated the antimicrobial efficacy of Triphala, green tea polyphenols (GTP), a mixture of tetracycline isomer, acid and detergent (MTAD), and 5% sodium hypochlorite (NaOCl) against *E. faecalis* biofilm on tooth substrates. Results showed that Triphala, MTAD, and NaOCl completely inhibited the three-week biofilm, while GTP and saline did not. For the six-week biofilm, only NaOCl showed complete inhibition, though Triphala, GTP, and MTAD still significantly reduced bacterial counts. The study concluded that NaOCl had the highest antibacterial activity, but Triphala, GTP, and MTAD also demonstrated significant efficacy, suggesting herbal alternatives might be beneficial as root canal irrigants due to NaOCl's undesirable characteristics. The three different compounds of Triphala help enhance its properties resulting in a synergistic antimicrobial effect.

**Figure 6:**
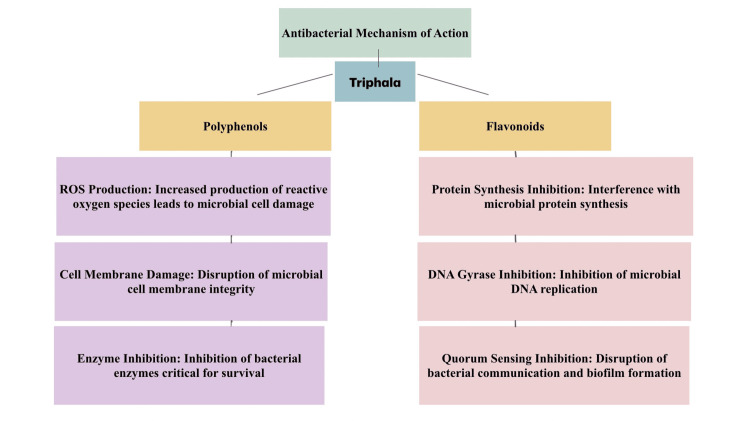
Mechanism of antimicrobial activity of Triphala Mechanism of synergistic effects of the antimicrobial activity exhibited by Triphala in terms of the individual components and their mode of action. ROS: reactive oxygen species Credits: Illustration created by Dr. Neha Kannan

**Figure 7:**
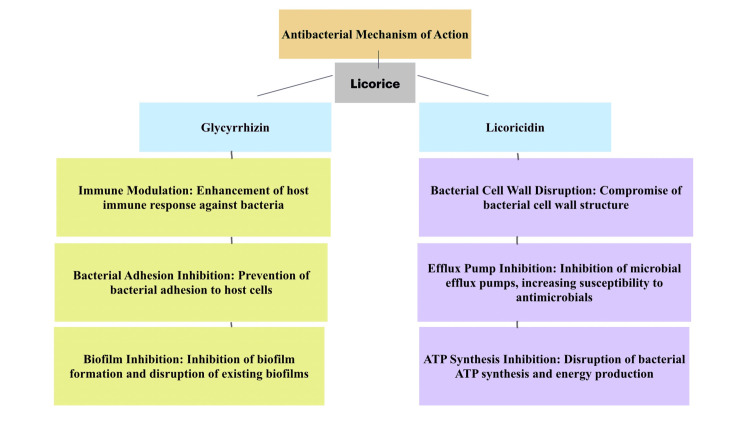
Mechanism of action of Licorice Mechanism of synergistic effects of the antimicrobial activity exhibited by Licorice in terms of the individual components and their mode of action. ATP: adenosine triphosphate Credits: Illustration created by Dr. Neha Kannan

By integrating these beneficial properties into a single gum paint formulation, this study opens new avenues for developing effective oral health treatments. This innovative approach not only leverages the known benefits of Triphala and Licorice but also explores their synergistic effects, potentially leading to more comprehensive and effective oral healthcare solutions (Figures 6, 7).

Clinical implications

**Figure 8:**
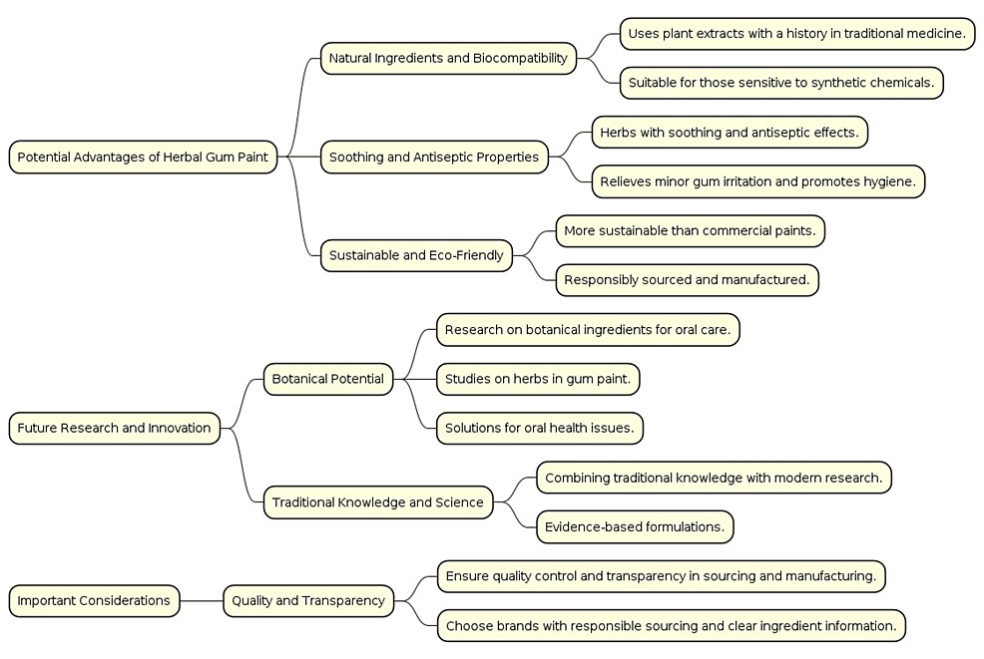
Clinical implications of the formulated herbal gum paint Flowchart delineating the clinical implications of the herbal gum paint in terms of sustainability, biocompatibility, quality control and transparency. Credits: Flow chart created by Dr. Neha Kannan

The study highlights the clinical implications of using a gum paint formulation made from Licorice and Triphala, emphasizing its potential to enhance oral health. The antimicrobial properties demonstrated in the study suggest that this gum paint can effectively reduce or prevent oral infections such as gingivitis and periodontitis. Additionally, utilizing natural ingredients like Licorice and Triphala provides a safer alternative to synthetic antimicrobial agents, potentially reducing side effects and promoting a more holistic approach to oral health care (Figure 8).

Another significant finding from the study is the reduced cytotoxicity of the formulation. This ensures that the gum paint is safe for use in the oral cavity, not harming human cells, and making it a viable option for long-term oral hygiene maintenance. The anti-inflammatory properties of both Licorice and Triphala further contribute to the formulation’s benefits, helping to manage gum inflammation and providing relief from symptoms associated with various oral diseases.

The gum paint formulation can be used in several ways. As a daily oral hygiene product, it can be incorporated into routines for individuals looking to prevent dental plaque formation and oral infections naturally. Dentists might prescribe this formulation as part of the treatment regimen for patients with gum diseases such as gingivitis and periodontitis, leveraging its antimicrobial and anti-inflammatory properties to enhance overall disease management.

Post-surgical care is another area where this formulation can be beneficial. It can help prevent infections and promote healing in patients who have undergone dental surgeries or procedures. Additionally, its soothing and antimicrobial properties make it suitable for managing and treating oral ulcers, providing relief and accelerating the healing process. The black seed-assisted synthesis of iron oxide nanoparticles has also been used for the treatment of infections [21,22].

For individuals sensitive to conventional chemical mouthwashes, this natural formulation offers an effective alternative, reducing the risk of adverse reactions while ensuring oral health is maintained. Furthermore, the findings of this study can inspire the development of other oral care products, such as toothpaste or mouth rinses, incorporating Licorice and Triphala, thereby expanding the range of natural oral care solutions available to consumers.

In summary, the gum paint formulation from Licorice and Triphala has significant potential for enhancing oral health through its antimicrobial and anti-inflammatory properties with minimal cytotoxicity. This makes it a safe and effective option for both preventive and therapeutic applications in dental care.

Limitations

The study focuses on specific microbial strains relevant to oral health, potentially overlooking broader pathogen variations. The evaluation was over a short-term period, limiting insights into long-term effects on oral microbiota and tissue safety. The preliminary nature of findings requires further validation in larger clinical trials.

## Conclusions

The use of herbal alternatives, such as gum paint, could be advantageous given the undesirable characteristics of chemically manufactured products. This herbal gum paint exhibits potent antimicrobial activity with minimal cytotoxicity, making it a promising alternative to established commercial gum paints. Further research and clinical trials are necessary to confirm its efficacy in treating periodontitis, oral ulcers, and halitosis.
